# Feasibility of Endoscopic Resection for Sessile Nonampullary Duodenal Tumors: A Multicenter Retrospective Study

**DOI:** 10.1155/2015/692492

**Published:** 2015-02-24

**Authors:** Sung Min Park, Joo Ho Ham, Byung-Wook Kim, Joon Sung Kim, Chang Whan Kim, Jin Il Kim, Chul Hyun Lim, Jung Hwan Oh

**Affiliations:** ^1^Division of Gastroenterology, Department of Internal Medicine, Incheon St. Mary's Hospital, College of Medicine, The Catholic University of Korea, Seoul 403-720, Republic of Korea; ^2^Bucheon St. Mary's Hospital, College of Medicine, The Catholic University of Korea, Seoul 403-720, Republic of Korea; ^3^Yeouido St. Mary's Hospital, College of Medicine, The Catholic University of Korea, Seoul 403-720, Republic of Korea; ^4^Seoul St. Mary's Hospital, College of Medicine, The Catholic University of Korea, Seoul 403-720, Republic of Korea; ^5^St. Paul's Hospital, College of Medicine, The Catholic University of Korea, Seoul 403-720, Republic of Korea

## Abstract

*Objectives*. Sessile nonampullary duodenal tumors (SNADTs) are relatively rare and endoscopic resection of these lesions is considered more challenging than in other parts of the gastrointestinal tract. The aim of this study was to evaluate the feasibility of endoscopic resection for SNADT. *Methods*. Medical records including endoscopic resection for SNADT from July 2002 to July 2013 from 5 centers affiliated to The Catholic University of Korea were reviewed retrospectively. Demographic features and clinical outcomes such as complete resection and complications were analyzed. *Results*. A total of 56 lesions from 54 patients were enrolled in this study. Forty-five lesions were resected by endoscopic mucosal resection (EMR), 6 lesions by endoscopic submucosal dissection (ESD), and 5 lesions by simple polypectomy. Histologic examination after endoscopic resection revealed adenocarcinoma in 2, low grade adenoma in 25, high grade adenoma in 11, and carcinoid tumor in 18 lesions. *En bloc* resection rates and histological complete resection rates were 78.6% (44/56) and 80.0% (28/35), respectively. Bleeding which required additional endoscopic intervention occurred in 1.8% (1/56) and perforation in 7.1% (4/56). There was no procedure-related mortality. *Conclusions*. Endoscopic resection techniques including ESD might be safe and effective modalities for the management of SNADT.

## 1. Introduction

Nonampullary duodenal tumors (NADTs) are reported in 0.3–4.6% of patients attending for upper gastrointestinal endoscopy [1–3]. Most of these lesions have been resected surgically since endoscopic intervention in the duodenum is related with a higher risk of complications compared to the treatment of premalignant lesions and early malignant lesions of the esophagus, stomach, and colorectum [4, 5].

Endoscopic management of NADTs provides a challenge in terms of accurate diagnosis, staging, and endoscopic resection in the presence of the thin duodenal wall and rich vascularity. However, endoscopic approach offers considerable advantages in terms of organ preservation, procedure-related risks, recovery, and length of hospital stay. There was a report that surgical or endoscopic resection of early duodenal cancer resulted in no lymph node metastasis in any of the cases among 128 lesions of intramucosal carcinoma [6]. These results advocate the rationale for endoscopic resection for NADTs.

Although there were some reports on the efficacy of endoscopic resection for NADTs from various single centers, multicenter studies have not been reported. The aim of this study was to evaluate the feasibility of endoscopic resection for the management of sessile NADTs (SNADTs) on multicenter basis.

## 2. Materials and Methods

### 2.1. Study Population

Medical records on endoscopic resection for SNADTs were reviewed in 5 teaching hospitals affiliated to The Catholic University of Korea (Incheon St. Mary's Hospital, Bucheon St. Mary's Hospital, Yeouido St. Mary's Hospital, Seoul St. Mary's Hospital, and St. Paul's Hospital) from July 2002 to July 2013. At least 50 cases of EMR and/or ESD for neoplasia of upper gastrointestinal tract per year are performed in every center. Patients with ampullary or periampullary lesions as well as patients with a history of familial polyposis syndromes were excluded. Pedunculated polypoid lesions were also excluded since these lesions can be easily removed by endoscopy. After reviewing the final pathologic reports acquired from endoscopic resection, adenoma, adenocarcinoma limited to the mucosal layer, and carcinoid tumors limited to the mucosa were included in this study. Demographic characteristics including sex and age and characteristics of the sessile lesions such as number, size, location, histologic findings, and endoscopic resection method were identified. The Institutional Review Board of The Catholic University of Korea approved this study.

### 2.2. Endoscopic Resection

The techniques of endoscopic resection were classified into three groups: endoscopic polypectomy (EP), which was performed by snare only without injection into submucosal layer; endoscopic mucosal resection (EMR), which was performed by snare after injection into submucosal layer; endoscopic submucosal dissection (ESD), which included the steps of precutting of mucosa and dissection of the submucosal layer with knives after injection into submucosal layer.

### 2.3. Definition of Terms and Endoscopic Treatment Outcomes

*En bloc* resection was defined as when a tumor was resected in one piece without fragmentation. Histological complete resection was defined as when lateral and deep resection margins were free of tumor after resection.

Bleeding was defined as intraprocedural massive bleeding that required blood transfusions or postprocedure bleeding that required blood transfusion, endoscopic intervention, or surgical intervention.

Perforation was defined when intra-abdominal space was directly observed during the procedure (frank perforation) or free air was found on a plain chest X-ray after procedure without a visible duodenal wall defect during procedure (microperforation).

Local recurrence was defined as identifying a microscopic adenoma and/or carcinoid tumor at the original tumor site during the follow-up period. Follow-up period was defined as the interval between the date of resection and the most recent endoscopic examination.

### 2.4. Statistics

Differences in overall outcomes among the endoscopic resection methods were evaluated using the Kruskal-Wallis test or Mann-Whitney *U* test for continuous data and *χ* ^2^ test for categorical variables. Statistical analyses were conducted using SPSS version 15.0 for Windows software (SPSS, Chicago, IL, USA) and *P* value less than 0.05 was considered statistically significant.

## 3. Results

### 3.1. Patients' Characteristics

**Figure 1:**
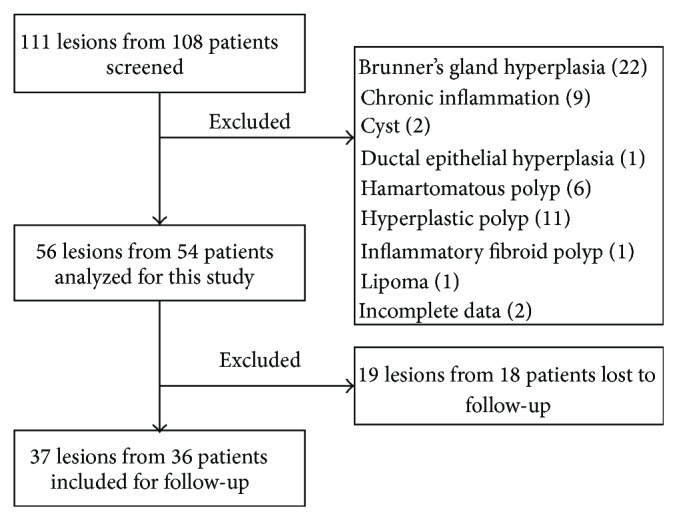


One hundred eleven lesions from 108 patients were screened and 56 lesions from 54 patients were identified (Figure 1). Thirty-seven lesions from 36 patients were analyzed for the follow-up study. Demographic features of the 56 lesions are shown in Table 1. Mean age was 59.5 ± 12.5 years and male to female ratio was 33 to 21. EP, EMR, and ESD were performed in 5 lesions, 45 lesions, and 6 lesions, respectively. Average follow-up period was 31.7 ± 21.5 months.

Most of the lesions were located in 1st and 2nd portion of duodenum in a single lesion. Mean length of the long axis was 1.06 cm. On histologic examination, adenoma with low grade dysplasia was found in 25 lesions (44.7%), adenoma with high grade dysplasia in 11 lesions (19.7%), adenocarcinoma in 2 lesions (3.6%), and carcinoid tumors in 18 lesions (32.1%).

### 3.2. Outcomes of Endoscopic Resection

Outcomes of endoscopic resection are summarized in Table 2.* En bloc* resection rate was 78.6% (44/56) and histological complete resection rate was 87.5% (49/56).

Immediate complication was described in 5 patients (1 bleeding and 4 perforations; Table 2). One case of bleeding occurred 12 hours after EMR and presented as hematochezia. This patient was successfully treated with injection of epinephrine mixture and with clips. Frank perforation occurred in 2 patients who underwent surgical management. Among 2 patients with microperforation, 1 patient was managed surgically and the other patient was managed endoscopically. There was no procedure-related mortality.

### 3.3. Long-Term Outcomes

Median follow-up period was 33.5 months for EP, 6.0 months for EMR, and 18.0 months for ESD. Among these patients, local recurrence occurred in 1 patient who was treated with EMR. In this patient, resection margin was positive and the recurrent lesion was found 2 months after resection. This recurrence was ablated with argon plasma laser coagulation and there was no recurrence after the ablation therapy for the following 18 months.

## 4. Discussion

In duodenum, primary epithelial neoplasia and carcinoid tumors are very rare, although their incidence has been increased in Korea for the past decade in part because of increased screening endoscopy and because of better awareness. Surgical and/or endoscopic resection is recommended for these lesions due to malignant potential of both lesions [7, 8]. However, surgical resection for duodenal tumors such as pancreaticoduodenectomy may be associated with perioperative morbidity, mortality, and long-term complications affecting the quality of life [9, 10]. Therefore, in recent years, the frequency of endoscopic treatment has been increasing to avoid mortality and morbidity of surgical treatment. Despite increasing frequency, there have been only few reports on the outcomes of endoscopic treatment for NADTs. Most of previous reports included a small number of patients and were performed in single centers [11–14]. We therefore performed this retrospective study on a multicenter basis.

Pedunculated lesions can be easily removed by EP technique and complications such as perforation might be extremely rare. So we analyzed sessile lesions only (Paris Classification Is and II lesions) [15] while previous studies included pedunculated lesions or did not describe the characteristics of the lesion [11–14]. We included carcinoid tumors of duodenum since carcinoid tumors are sessile in most cases and can be managed equivocally with other types of epithelial neoplasia such as adenoma and adenocarcinoma [16, 17]. Considering that carcinoid tumors originate from enterochromaffin-like cells which are one of the epithelial lining cells of gastrointestinal tract, they are worth being considered together.

In this study, EP seemed to be superior compared to other techniques in the point of complete resection and complications. However, EP can only be performed in lesions with a good view and when the size is small enough to be removed without submucosal injection. ESD required statistically significant more procedure time compared to EP and EMR. However, it is plausible that ESD might be selected as a procedure of choice when* en bloc* resection by other techniques is expected to be difficult. There was no difference in* en bloc* resection rates among these procedures. Considering the learning curve for ESD [18, 19],* en bloc* resection rate of ESD is expected to be increased.

Although EMR was the most commonly performed procedure and other techniques such as EP and ESD were limitedly performed in this study, overall outcomes of endoscopic resection for SNADTs were quite favorable.* En bloc* resection rate was 78.6% (44/56) and histologic complete resection was achieved in 83.9% (47/56) in total, which was comparable with previous single center studies [11–14]. Procedure-related complications such as bleeding (1.8%) and perforation (7.8%) were not common in this study as in other previous studies [11–14] and were managed successfully. There was no procedure-related mortality.

There are some limitations in this study. Although our study was a multicenter study, its retrospective design may have resulted in underreporting of complications and selection bias might have occurred inevitably. We tried to compare each endoscopic technique but patients who underwent EP and ESD were relatively small in number compared to EMR. Follow-up endoscopy was arbitrarily performed and follow-up was not evenly performed in some patients.

In conclusion, endoscopic resection for SNADT seems to be effective and safe. Additional studies including large number of cases and prospective design with long-term follow-up is anticipated.

## Figures and Tables

**Table 1:** Demographic features.

Number of patients	54
Number of lesions	56
Mean age (years ± SD^*^)	59.5 ± 12.5
Male : female (%)	33 : 21 (61.1 : 38.9)
Histologic types (%)	
Adenoma, low grade dysplasia	25 (44.6)
Adenoma, high grade dysplasia	11 (19.6)
Adenocarcinoma	2 (3.6)
Carcinoid tumor	18 (32.1)
Location (%)	
1st portion	24 (42.9)
2nd portion	31 (55.4)
3rd portion	1 (1.8)
Size of the lesions (median (range), cm)^†^	
EP	1.2 (1.0~1.5)
EMR	0.8 (0.3~4.5)
ESD	0.8 (0.4~3.5)

^*^SD: standard deviation.

^†^Size of long axis.

EP: endoscopic polypectomy; EMR: endoscopic mucosal resection; ESD: endoscopic submucosal dissection.

**Table 2:** Outcomes and complications of endoscopic resection.

	EP (*n* = 5)	EMR (*n* = 45)	ESD (*n* = 6)	*P* value
*En bloc* resection (%)	5 (100)	35 (77.8)	4 (66.7)	0.414
Histologic complete resection (%)	5 (100)	37 (82.2)	5 (83.3)	1.000
Procedure time (median (range), min)	5.0 (5~16)	13.0 (10~130)	41.5 (32~180)	0.003
Complications (%)	0 (0)	3 (6.7)	2 (33.3)	0.140
Bleeding (%)	0 (0)	1 (2.2)	0 (0)	1.000
Perforation (%)	0 (0)	2 (4.5)	2 (33.3)	0.088
Follow-up period (median (range), mon)	33.5 (7~60)	6.0 (3~66)	18.0 (2~96)	1.000
Recurrence rate (%)^*^	0/2 (0)	1/29 (3.4)	0/5 (0)	0.632
Number of cases and complications in each center†				
Incheon St. Mary's Hospital	0	4 (0)	2 (1)	
Bucheon St. Mary's Hospital	2 (0)	10 (2)	1 (0)	
Yeouido St. Mary's Hospital	0	15 (0)	3 (1)	
Seoul St. Mary's Hospital	3 (0)	15 (1)	0	
St. Paul's Hospital	0	1 (0)	0	

EP: endoscopic polypectomy; EMR: endoscopic mucosal resection; ESD: endoscopic submucosal dissection.

^*^Recurrence rate was obtained from patients who were followed up at least 2 months.

^†^Numbers indicate the total number of procedures and the numbers in parentheses indicate the number of complications.
